# Medical Science at Perth

**Published:** 1887-10-22

**Authors:** 


### The Hospital

A Weekly Institutional Journal of

Science, Medicine, and Philanthropy.

*For Hospitals, Asylums, Medical Practitioners, Students, Nurses, Families, Parishes, Congregations, and the Charitable Public.*

Vol. III. No. 56.] [October 22, 1887.

### Medical Science at Perth.

Travellers to the Northern Highlands will remember the Perth Railway Station, and the excellent breakfast of salmon steak, ham and eggs, "rolls" and fresh butter, with jams, marmalades, and the hottest of hot coffee which awaits them there on a chill August morning after a twelve hours' run from London. Little boys at Board-schools are able to inform us that the city of Perth is the principal town of the county of that name; that it is situated on the river Tay, not far from the busy commercial town of Dundee, and less than three hours by rail from those centres of learning, law, theology, and business—Edinburgh and Glasgow. Those who are familiar with the city know that it is of ancient fame, and boasts of having in its near vicinity the ruins of the Palace of Scone, where Scottish kings in times by no means remote held sway and rule. It contains a population of nearly thirty thousand; sends a representative to Parliament; has fairs and markets; churches by the dozen, of all denominations; a corporation and a provost, a beadle and a bellman, a prison and a town-pump. Add to this that there is an infirmary and a fever hospital; five-and-twenty medical men in full practice; a famous "Academy" with a competent staff of teachers, and innumerable schools of the second and third degree,—and it will be seen that neither by distance from centres of civilisation nor by dearth of the means of learning and culture ought Perth to be a place of second-rate civilisation, walking in the light of a science long since exploded, and a practice out of date, even among village apothecaries.

But such appears to be the fact. A story comes to us through the columns of *The Dundee Advertiser*, which if true ought to make every first year's medical student blush for shame. These are the facts as stated by the *Advertiser*. According to the report of Dr. Simpson, the Medical Officer of Health for the city, small-pox broke out in the Perth Infirmary in the early part of the present month. The first case came to light on the 4th, and two other cases were subsequently ascertained. On the night of the 4th—that is, on the very day that the diagnosis was made certain—the Sanitary Inspector received a peremptory order from the Infirmary medical staff to open the small-pox hospital, and to take the three patients in at once, on the double ground that there was no room to treat them at the general hospital, and that the spread of infection could not be prevented there. So far, well! The staff acted with excellent promptitude, and on thoroughly scientific lines. Within a few days, however, three of the Infirmary nurses were attacked with small-pox, and of course it was expected that they also, on precisely the same grounds, would have been removed to the small-pox hospital. But what actually occurred? The Infirmary authorities declined to allow the nurses to be taken to the small-pox hospital—first, because they had now found room for them at the Infirmary; and secondly, because they were ladies, and the small-pox hospital was not adapted for the reception of ladies. A third point should have been added, which was apparently forgotten, that small-pox in ladies is not infectious, as it is in poor persons, and that therefore the other patients in the Infirmary could not come to any harm. At the time of writing, the three nurses are still suffering from small-pox, still under treatment at the Infirmary where the other patients are; and a fresh case of the disease is reported among the inmates.

Now we have to ask, Who is responsible for this condition of affairs? Is it some obstinate member of the Committee, who knows as much of sanitary science as Moses did of Shakespeare; or some superannuated Baillie, who takes his medical knowledge from the Catechism, or from Boston's *Fourfold State*? Will it be believed that the directors of the Infirmary were the responsible persons, and that "they had the benefit" (*sic*) "of the doctors of the Infirmary, and also the senior consulting physician and surgeon"? Dr. Stirling, says the Lord Provost, "being asked his opinion, distinctly stated that there was not one particle of possibility of infection being carried beyond the ward in which these patients were isolated; that there was not a scintilla of danger; and further, that there might be danger in removing the patients at that particular time." That is to say—on the 4th, when three patients were attacked, instant removal was considered imperative by the medical staff, on the ground, among others, that the spread of infection could not be prevented at the Infirmary. A few days later, when three nurses are attacked, the same medical staff consider that there is not "a particle of possibility of danger"; not even "a scintilla," on the excellent and unquestionable authority of Dr. Stirling.

Who, then, is Dr. Stirling, and whence comes this absolute scientific infallibility—an infallibility not merely of knowledge but of power? For does he not say one day that infection cannot be prevented at the Infirmary, and another that there is not a "particle of possibility of danger"? Dr. Stirling is M.D. of the University of Edinburgh; he graduated in 1853; is consulting medical officer to the County and City of Perth Infirmary; and a literary contributor on "Leprosy in Norway." Clearly, then, he is a man of some age and experience, with a high-class qualification, and an honourable professional status: a man whom the public have delighted to honour, and on whose scientific veracity men are accustomed to rely. We have a question to ask him: Does he or does he not know that there is not a "particle of possibility" of infection "being carried beyond the ward in which small-pox patients are lying"? And if he knows, when and where has he acquired the knowledge; and how is he going to set about converting the whole medical and scientific world, whose opinions are as diametrically opposite to his as the North Pole is to the South?

Is Dr. Stirling one of those dangerous men who wish a thing, and straightway the thing is exactly as they wish? Did he ever hear the Scottish proverb, "Facts are chiels that wunna ding, and daurna be disputed"? Dr. Stirling, like the late Dr. Begg, familiar to all Scotchmen, evidently thinks that facts are "chiels" that will "ding," and that "maun ding" if he makes up his mind to it. That habit of mind was all very well in the regions of a metaphysical theology, where Dr. Begg and his like could believe what they pleased without injury to anybody but themselves. But we submit that when such principles are brought into medical practice, and a whole infirmary full of people are exposed to imminent danger of small-pox because one unscientific Athanasius chooses to defy the world, the case wears quite a different aspect. It is then no time for amused laughter over the morning *Scotsman*, but for prompt and competent inquiry on the part of unprejudiced scientists, in order that those poor persons who have placed themselves under the care of the authorities of the Perth Infirmary may no longer be exposed to the risks of danger and death.

We counsel the Perth people, and especially the directors of the Infirmary, to put an end to this dangerous and utterly unjustifiable state of things without an hour's delay. If they cannot rely upon the judgment and experience of their Medical Officer of Health, there are men of the first eminence in Edinburgh, Glasgow, and other towns who should be consulted forthwith. It is absolutely unpardonable that a whole infirmary and a whole town should be exposed to so terrible a calamity as small-pox because one wrongheaded man leads a feeble clique to defy the veriest rudiments of medical and sanitary science. Athanasius *contra mundum* may have been sublime: Dr. Stirling *contra scientiam* is entirely ridiculous.

